# Gyroidal graphene/porous silicon array for exciting optical Tamm state as optical sensor

**DOI:** 10.1038/s41598-021-98305-0

**Published:** 2021-09-29

**Authors:** Zaky A. Zaky, Arafa H. Aly

**Affiliations:** TH-PPM Group, Physics Department, Faculty of Science, Beni-Suef University, Beni-Suef, Egypt

**Keywords:** Photonic crystals, Optical sensors, Terahertz optics, Biosensors, Nanophotonics and plasmonics

## Abstract

In this study, the optical Tamm state is excited for the first time using gyroidal graphene/porous silicon one-dimensional photonic crystal terminated by a gyroidal graphene layer. The gyroidal graphene and porous silicon are used to enhance the figure of merit and sensitivity of the based Tamm resonance photonic crystal sensor. By tuning different parameters like the angle of incidence, the thickness of the sample layer, and the thickness of the gyroidal graphene layer, we have reached the optimized sensor. The observation of resonant dips in the reflectance spectra is strong evidence that Tamm plasmon-polaritons exist with higher sensitivity (188.8 THz/RIU) and figure of merit (355,384 RIU^−1^) than previously reported structures. The proposed sensor recorded sensitivity and FoM higher 38% and 747% respectively than a similar structure composed of graphene sheets and porous silicon.

## Introduction

Tamm plasmon-polariton (TPP) is an optical resonance excited at the interface between metal and a periodic one-dimensional photonic crystal (1D-PC)^1–9^. Contrary to the conventional surface plasmon resonance, TPP can be excited for both electromagnetic polarizations, at any incident angle, and without using grating or a coupling prism^10^. Besides the fact that the incident electric field is localized within the defected 1D-PC of the structure^11–15^, porous layers filled with analyte samples will be proposed in this study^3,16^. Recently, we proposed the excitation of TPP by porous silicon PC (PSi-PC) using graphene sheets^17^ or silver^16^. Porous silicon multilayers can be experimentally prepared by electrochemical etching with hydrogen fluoride as an electrolyte that is considered a simple one-step fabrication^18,19^.

In 2017, Bikbaev et al. investigated a TPP excited by metallic gyroid layer^20^. In 2020, Sun et al. fabricated a gyroid structure of bioinspired Au–CuS to excite SPR^21^. In these calculations, we will use gyroidal graphene (GGr)/porous silicon array for the first time to excite TPP. The gyroidal structure possesses a surface with fixed mean curvature within a volumetric fill fractions range^22^. Gyroidal structures can be experimentally realized using inorganic templates from the butterfly nanostructure or self-assembly^23^, chemical vapor deposition^24^, self-assembly of a triblock copolymer^25^, selective laser melting^26^, solvent-free method^27^, light-based 3D printing process^28^, and controlled phase separation^29^. Graphene has very distinguished optical, electrical, and mechanical properties^6,30^. Chemical doping or the use of an external gate voltage can change the carrier concentration of graphene layers^31^. The negative conductivity of graphene at a certain frequencies makes it behave as a metalic layer^32–36^. So, the gyroidal graphene layer will be used to excite Tamm states.

## Materials and theoretical method

**Figure 1:**
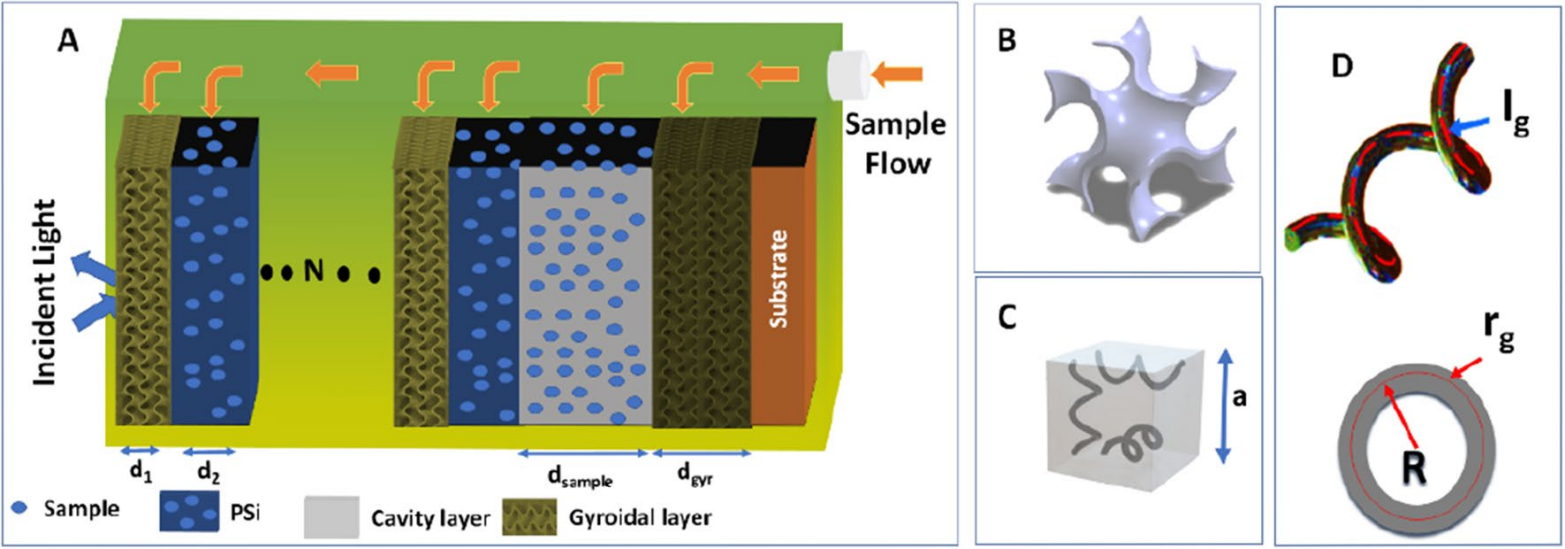
**(A)** The schematic structure of the gyroidal graphene PSi-PC, **(B)** gyroidal model^37^, **(C)** unit cell of tri-helical gyroid^20^, and **(D)** helix geometrical sizes^20^. For **(B–D)** (reprinted/adapted) with permission from ref.^37^ and^20^ respectively.

The proposed structure will be (GGr_1_/PSi)^N^/cavity/GGr_2_/ substrate as clear in Fig. 1A. N represents the number of PC periods. The optical permittivity of the graphene gyroid layer (Fig. 1B) will be calculated according to the following model^20,38^:

1 $${\upvarepsilon }_{gyr}(\upomega )=\frac{{\mathrm{l}}_{g}\sqrt{2}}{a}\left[1-{\left(\frac{{4\mathrm{r}}_{g}}{{\lambda }_{g}}\right)}^{2}{\left(\frac{\pi \sqrt{-{\upvarepsilon }_{m}(\upomega )}}{2\sqrt{2}n}-1\right)}^{2}\right],$$

where a,${r}_{g,}f, {\lambda }_{g}, {l}_{g}$ are the gyroidal unit cell size (Fig. 1C), helix radius, the volume fraction of graphene in the gyroidal layer, effective plasmon frequency, and the wire turn length. The length of the wire ring (Fig. 1D) is calculated as:

2 $${\mathrm{l}}_{g}=\sqrt{{\left(2\pi R\right)}^{2}+{a}^{2}}=a\sqrt{\frac{{\pi }^{2}{\left(\sqrt{2}-1\right)}^{2}}{4}+1},$$

R, ${\mathrm{r}}_{g}$ and ${\lambda }_{g}$ can be calculated as:

3 $$\mathrm{R}=\frac{{\left(\sqrt{2}-1\right)}^{2}}{4}+1,$$

4 $${\mathrm{r}}_{g}=\mathrm{a}\sqrt{f}\frac{\sqrt[4]{2}}{\sqrt{\pi \left[\sqrt{2+{\pi }^{2}}+\sqrt{2+\left(3+2\sqrt{2}\right){\pi }^{2}}\right]}},$$

5 $${\lambda }_{g}=1.15{\mathrm{a}}\sqrt{1-0.65lnf}.$$

According to the approximate local random phase, the conductivity of monolayers of graphene (σ) in the THz frequency range is calculated as^17,39,40^:

6 $$\begin{array}{ll}\upsigma & ={\mathrm{G}}\frac{i {e}^{2}{\mathrm{ E}}_{\mathrm{F}}}{\uppi {\mathrm{\hslash }}^{2}(\omega +\frac{i}{\tau })}\end{array},$$

where G, E_F_, e, ω, τ are the graphene sheets’ number, Fermi energy, the charge of the electron, angular frequency, and the relaxation time of the graphene sheet. Bruggeman’s effective medium equation is used to calculate the optical refractive index of the PSi layer (n_PSi_ ) filled with sample (n_sample_ ) with porosity (P)^41,42^:

$${\mathrm{n}}_{\mathrm{Psi}}^{ }=0.5\sqrt{\uppsi +\sqrt{{\uppsi }^{2}+8 {\mathrm{n}}_{\mathrm{si}}^{2} {\mathrm{n}}_{\mathrm{sample }}^{2}}},$$

7 $$\uppsi =3\mathrm{ P }\left({\mathrm{n}}_{\mathrm{sample }}^{2}-{\mathrm{n}}_{\mathrm{si}}^{2}\right)+\left(2 {\mathrm{n}}_{\mathrm{si}}^{2}-{\mathrm{n}}_{\mathrm{sample }}^{2}\right).$$

The refractive index of silicon ($${n}_{Si})$$ in THz range is 3.42^43^. The transfer matrix method (TMM) is used to study the reflectance spectra of electromagnetic waves (TM mode) from the proposed structure as the following^44–47^:

8 $$\left(\genfrac{}{}{0pt}{}{{E}_{0}}{{H}_{0}}\right)={\prod }_{k=1}^{n}{a}_{k} \left(\genfrac{}{}{0pt}{}{{E}_{n}}{{H}_{n}}\right)= \left[\begin{array}{cc}{A}_{11}& {A}_{12}\\ {A}_{21}& {A}_{22}\end{array}\right]\left(\genfrac{}{}{0pt}{}{{E}_{n}}{{H}_{n}}\right)=A\left(\genfrac{}{}{0pt}{}{{E}_{n}}{{H}_{n}}\right).$$

The ${H}_{n}$ and ${E}_{n}$ are the total magnetic and electric fields at the end of the proposed structure with k number of layers, ${H}_{0} \,{\mathrm{and}}\, E_{0}$ are total magnetic and electric fields at the air, and ${a}_{k}$ is the characteristic matrix of each layer of the proposed structure (k = 1 to n). A is the total TMM.

The characteristic matrix of each layer can be calculated as follows:

9 $${a}_{k}=\left(\begin{array}{cc}cos{\mathrm{\varnothing }}_{k}& -\frac{i}{{p}_{k}}sin{\mathrm{\varnothing }}_{k}\\ -i{p}_{k}sin{\mathrm{\varnothing }}_{k}& cos{\mathrm{\varnothing }}_{k}\end{array}\right),$$

where phase difference (${\varnothing }_{k}$) is:

10 $${\mathrm{\varnothing }}_{k}= \frac{2\pi {n}_{k}{d}_{k}cos{\varphi }_{k}}{\lambda },$$

where ${n}_{k},$ ${\varphi }_{k}$ and ${d}_{z}$ are the index of refraction, incident angle, and thickness of each layer. The following is the light reflectance of the suggested sensor:

11 $$R\left(\%\right)=100*{\lfloor r \rfloor}^{2},$$

where r is the reflection coefficient.

### Ethics declarations

This article does not contain any studies involving animals or human participants performed by any of the authors.

## Results and discussions

The thicknesses of layers GGr_1_, PSi, cavity, GGr_2_ are 90 nm, 4500 nm, 12,000 nm, and 400 nm, respectively. Besides, the filling factors of GGr_1_ GGr_2_, PSi are 3%, 20%, and 50%, respectively. The space areas inside the layers GGr_1_, GGr_2_, PSi, and cavity layer will be filled with the gas sample (n_sample_). The unit cell size of gyroid (a) is 30 nm, ${E}_{F}=1$ eV, $\tau =1 Ps$, and G = 1 (monolayer of graphene).

**Figure 2:**
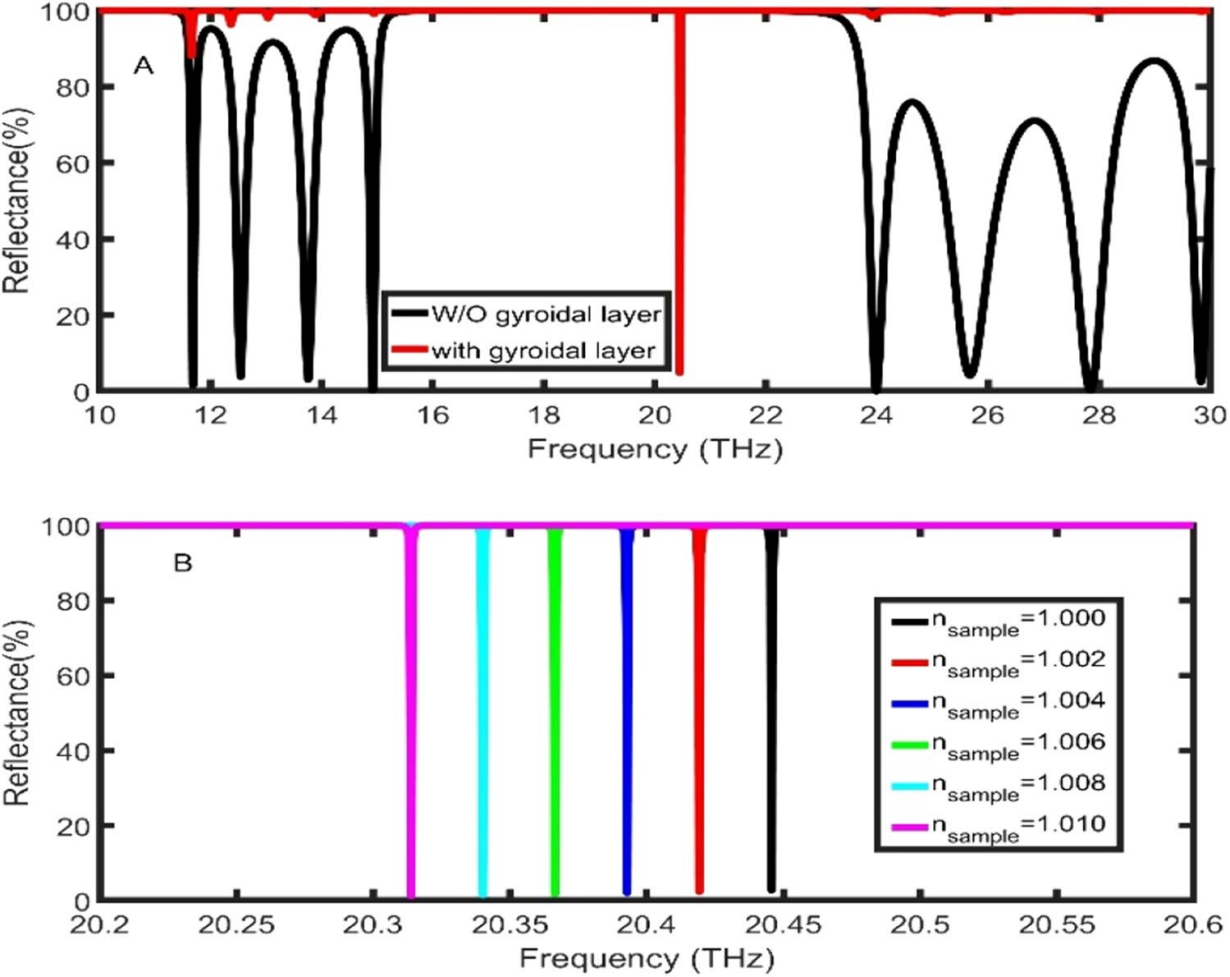
The reflectance spectra of the proposed PSi-PC structure **(A)** without GGr (black line) and with GGr (red line) at nsample = 1.00, **(B)** with GGr at different values of sample layer index of refraction.

As clear in Fig. 2A, the proposed structure without GGr_2_ possesses a photonic bandgap (PBG) with a bandwidth of 7.53 THz and 100% reflectance. By adding the GGr_2_ layer as a plasmonic layer, the TPP dip appears inside the frequency range of the PBG at 20.45 THz and 4.8% reflectance. Appearing of TPP dip is due to the trapping of light at the interface as a result of the PBG in the PSi-PC and strong attenuation of electromagnetic waves in graphene.

As the position of the TPP dip is strongly dependent on the effective refractive index of the whole structure, the resonant dip can be tuned with any change in the refractive index of the analyte inside the pores of the layers. As clear in Fig. 2B, by increasing the refractive index of the gas sample from 1.00 to 1.01, the TPP dip is red-shifted from 20.45 to 20.31 THz. Many parameters such as the sensitivity (S), and the figure of merit (FoM) can show the sensor performance as the following^48,49^:

12 $$S=\frac{\Delta {f}_{R}}{\Delta {n}_{s}},$$

13 $$FoM=\frac{S}{FWHM},$$

where $\Delta {f}_{R}$, $\Delta {n}_{s}$, and FWHM are the average change in TPP dip position, the average change in the sample refractive index, and the bandwidth of the TPP dips, respectively. At these calculations, the proposed sensor records sensitivity of 13.19 THz/RIU and FoM of 4.5 × 10^4^ RIU^−1^. To enhance these values of parameters, the effect of the incident angle, the thickness of the sample layer, and the thickness of GGr_1_ on the performance will be numerically studied.

**Figure 3:**
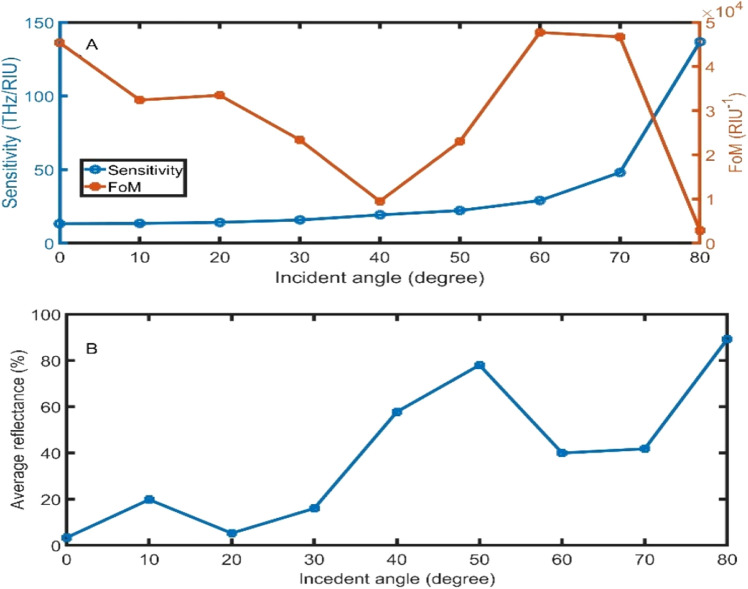
The **(A)** sensitivity and FoM, and **(B)** average reflectance of the proposed sensor as a function of the incident angle by changing the sample refractive index from 1.00 to 1.01.

Changing the incident angle is an effective way to control and enhance the performance of the TPP sensors^16,17^. The reflectance of experimentally PC was studied at the angle of incidence range from 0° to 90° in many works^50,51^. Within the proposed structure, the optical path length of the electromagnetic wave increases when the incidence angle increases. In addition, the confinement of the electromagnetic waves within the analyte increases^16^. Due to the previous results, the sensitivity increases from 13.19 THz/RIU to 136.71 THz/RIU with the increase of incident angle from 0° to 80° as clear in Fig. 3A. For angels higher than 80°, the resonant dip disappears. Besides, the FoM records the highest values at angles 0°, 60°, and 70°, and the lowest values at 40° and 80°. Figure 3B describes the average reflectance of the two resonant dips (at n_sample_ = 1.00 and n_sample_ = 1.01). Even though the angle of 80° has a high average reflectance (89%), the angle of 80° will be the optimum angle to achieve the highest sensitivity.

**Figure 4:**
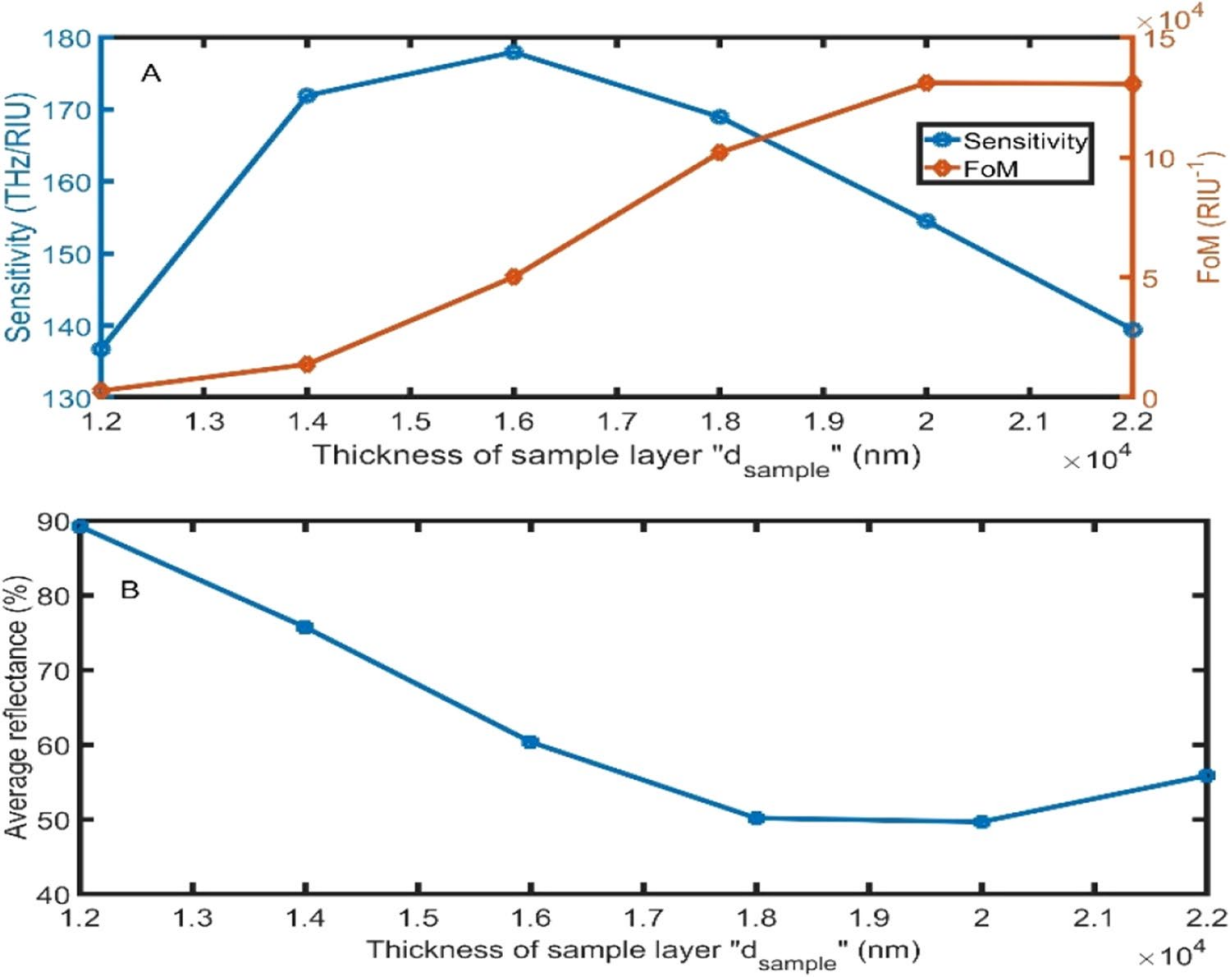
The **(A)** sensitivity and FoM, and **(B)** average reflectance of the proposed sensor as a function of the sample layer thickness by changing the sample refractive index from 1.00 to 1.01 at 80°.

As clear in Fig. 4A, the sensitivity of the gyroidal graphene PSi-PC increases from 136.71 THz/RIU to 177.95 THz/RIU with increasing the thickness of the sample layer thickness from 1200 to 16,000 nm. But the sensitivity decreases to 139.44 THz/RIU with increasing the thickness of the sample layer to 22,000 nm. In addition, the FoM rapidly increases from 284**8** to **131,052** RIU^−1^ with increasing the thickness from 12,000 to 20,000 nm. Further increases in the sample thickness will have a slight effect on the FoM. For the average reflectance, it decreases from 89 to 50% with increasing the thickness of the sample layer from 12,000 nm to 20,000 nm. Then, average reflectance increases for further thickness increase, as clear in Fig. 4B. We have strongly recommended the optimum thickness of 16,000 nm because it recorded the highest sensitivity.

**Figure 5:**
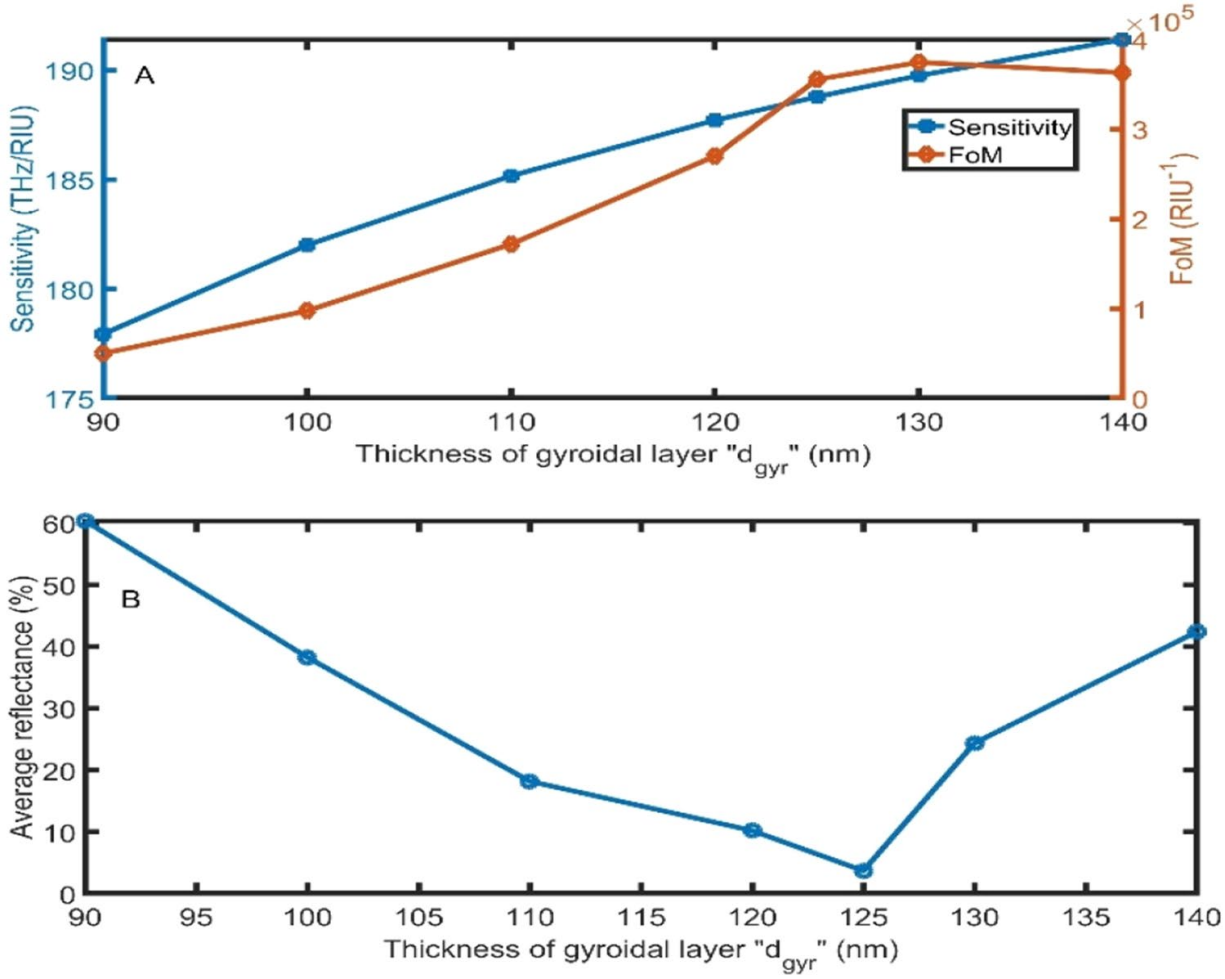
The **(A)** sensitivity and FoM, and **(B)** average reflectance of the proposed sensor as a function of the gyroidal graphene layer thickness by changing the sample refractive index from 1.00 to 1.01 at 80° and d_sample_ = 16,000 nm.

Herein, the effect of the gyroidal layer thickness (GGr_1_) is investigated, as clearly visible in Fig. 5. With the increase in the gyroidal layer thickness from 90 to 125 nm, both for sensitivity and FoM, the performance of the proposed sensor is dramatically enhanced to record sensitivity of 188.78 THz/RIU and FoM of 355,384 RIU^−1^, as clear in Fig. 5A. Even though the thickness of 125 nm does not record the highest values of sensitivity and FoM, it will be the optimum thickness of the gyroidal layer of graphene because it has the lowest reflectance, as clear in Fig. 5B.

**Figure 6:**
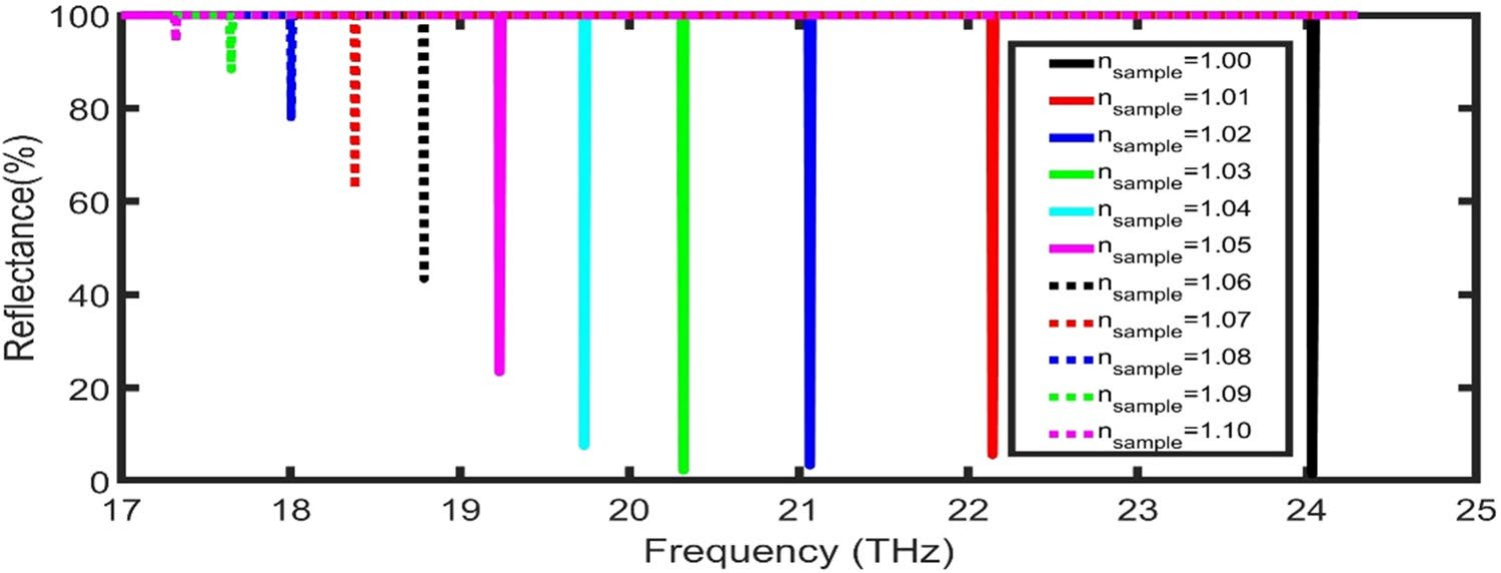
The reflectance spectra of the proposed sensor at different values of refractive indices.

As clear in Table 1, at different values of refractive indices, the Sensitivity and FoM will be studied for the proposed sensor. By increasing n_sample_ from 1.01 to 1.10, the sensitivity and FoM decrease from 188.76 THz/RIU to 33.00 THz/RIU and from 635,984 to 7535 RIU^**−**1^, respectively. As clear in Fig. 6, the highest performance was recorded at low values of n_sample_. Besides, with the increase of refractive index value, the reflectance of the TPP dips decrease. So, the proposed structure can be used in gas sensing and air pollution applications in refractive index range from 1.00 to 1.01.

**Table 1:** Sensitivity and FoM at different ranges of n_sample_:

n_sample_	S (THz/RIU)	$\mathrm{FoM}$ (RIU^−1^)
1.00	**–**	–
1.01	188.76	635,984
1.02	107.81	376,616
1.03	74.58	241,547
1.04	58.69	163,578
1.05	49.95	108,933
1.06	44.52	70,880
1.07	40.75	44,011
1.08	37.79	25,843
1.9	35.24	14,329
1.10	33.00	7535

**Figure 7:**
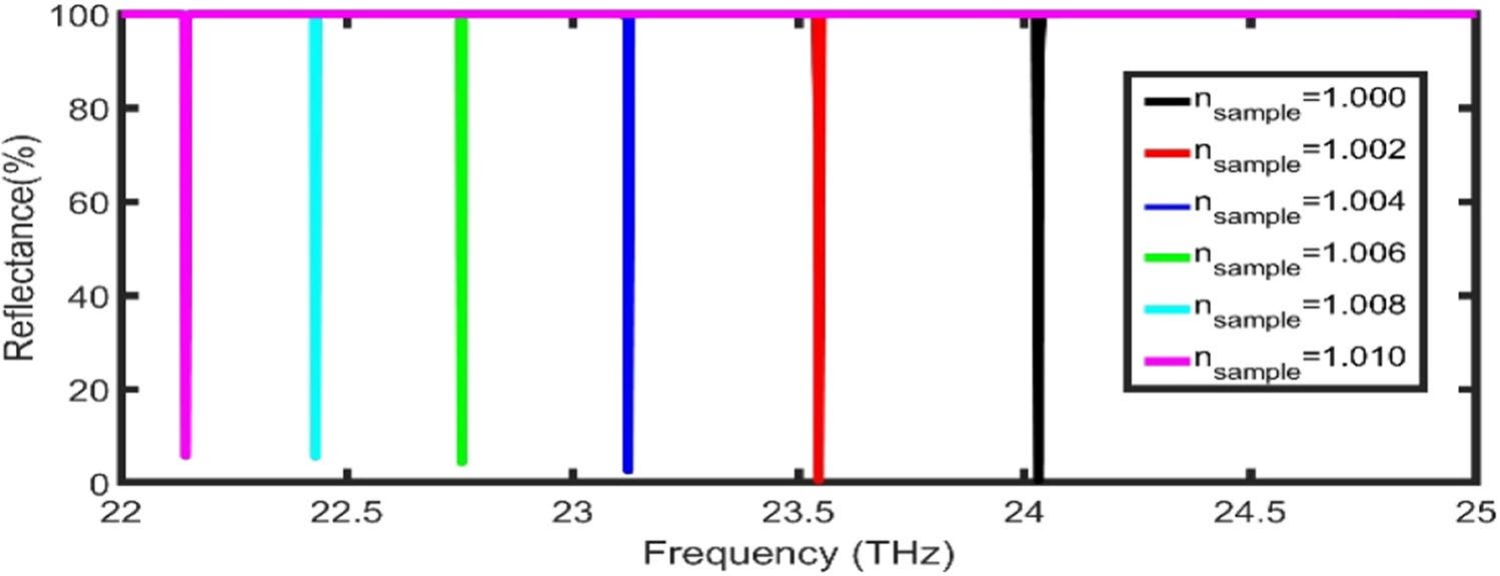
The reflectance spectra of the proposed sensor at the selected geometrical parameters.

Finally, in Fig. 7, the reflectance spectra of the proposed sensor at the selected geometrical parameters are plotted as a function of frequency. The sensitivity and FoM are much larger than many published papers, as clear in Table 2.

**Table 2:** Comparison with other designs performance (*NC* not be calculated).

Reference	S (THz/RIU)	FoM (RIU^−1^)	Refractive index range	Materials
^36^, 2019	0.7	10.3	1.00 to 1.10	Tamm with graphene
^52^, 2020	0.9	NC	1.15 to 1.76	Dirac semimetal
^53^, 2020	1.0	NC	1.15 to 1.76	Graphene-metastructure
^54^, 2020	0.3	8.4	1.00 to 2.44	Metamaterial
^55^, 2020	0.5	12.4	1.35 to 1.49	Dielectric metasurface
^56^, 2020	1.6	24.5	1.00 to 1.80	Graphene disks
^57^, 2020	1.7	7	1.00 to 2.00	Graphene metamaterials
^58^, 2020	1.0	14.1	1.00 to 2.20	Stacked metamaterials
^17^, 2021	4.75	475	1.00 to 1.72	Graphene and porous silicon
Our work	188.8	355,384	1.00 to 1.10	Graphene gyroid and porous silicon

TPP is shifted to lower or higher frequencies with a tiny change in the effective index of refraction of the whole geometrical structure or the medium that surrounds the structure^5^. The effective index of refraction of the whole geometrical structure is the summation of the index of refraction of each layer multiplied by the volume fraction of this layer in the structure. So, we have tried to increase the volume fraction of the sample in the structure by using a wide sample layer. Besides, we used porous silicon and gyroidal graphene filled with sample analyte. This design helped make any tiny change in the sample refractive index strongly affect the value of the effective refractive index of the structure and increase the TPP dip shift.

## Conclusion

A novel structure of 1D-PC of porous silicon/gyroidal graphene terminated with a gyroidal graphene layer was proposed for sensing applications in the THz range. The effect of the incident angle and thickness of the gyroidal and sample layer was studied. The proposed sensor recorded sensitivity and FoM higher 38% and 747% respectively than a similar structure composed of graphene sheets and porous silicon. Another benefit of the suggested design is that it can be used at room temperature. Besides, our sensor has ultra-high performance comparing with other published papers. This study provides a new train of investigated methods for designing nanophotonic devices based on porous silicon and gyroidal graphene layers.

## Data Availability

Requests for materials or code should be addressed to Z.A.Z.
